# 

**Published:** 1916-01-15

**Authors:** 


### CONTENTS.

| | PAGE |
|---|---|
| **The Profession and Titular Distinctions** ... | 335 |
| **The Whistling Nuisance** ... ... ... ... | 336 |
| **Hospital and Institutional News** ... ... ... | 337 |
| Hospital Repairs and Maintenance—Gifts in Memory of the Killed in Action—An Indefensible Practice—The Addington Park War Hospital—Experiences of a Captured Officer of the R.A.M.C.—The Western Hospital, Torquay—The Indian Army Corps Hospitals—The Lack of Isolation Hospitals in Cornwall—First-Aid for Street Accidents—Coal Supplies at Infirmaries—The Late Sir Frederic Hewitt—The Green Cross—Endowed Beds at Cardiff Hospital—The Recreation Room of the Future—A New Medical Playwright—The Organisation of Women Subscribers—Praise for the Stretcher-Bearers—Honour for Ex-Hospital Chairman—A Costly Alteration—A Grand Old Yacht—The British Women's Hospital—An Active Service Exhibition — The Serbian Retreat — Public Health and the Price of Land—The Private Patient in New Zealand—Anti-Toxin and the Fire Brigade—This Week's Drug Market. | |
| **A Medical Causerie** ... ... ... ... ... | 343 |
| **Patriotism in British Hospitals** ... ... ... | 344 |
| III.—The Royal Infirmary, Edinburgh. | |
| **An Important Military Research** ... ... | 345 |
| The Control of Bilharzia Disease. | |
| **The Treatment of Nævi** ... ... ... ... | 346 |
| **Our Voluntary Hospitals** ... ... ... ... | 347 |
| Some General Effects of the War. | |
| **Hospital Architecture and Construction** ... | 348 |
| Edith Cavell Home for Nurses : London Hospital. | |
| **The Heating of Hospitals** ... ... ... ... | 349 |
| VI.—The New King's College Hospital at Denmark Hill. | |
| **Mothercraft and Child-Welfare Centres** ... | 351 |
| II.—Difficulties and Problems. | |
| **Round the World** ... ... ... ... ... | 352 |
| **Hospital Repairs and Maintenance** ... ... | 353 |
| The Engineer's Department at Guy's Hospital. | |
| **Parliament and the Profession** ... ... ... | 354 |
| **The Editor's Letter-Box** ... ... ... ... | 355 |
| **Passing Events and Topics** ... ... ... | 356 |
| **The Institutional Worker** ... ... (Suppl.) | 1 |
| A Charity Appeal in War Time. | |
| Value of Fire Drill. | |

